# NF-kappa B Signaling-Related Signatures Are Connected with the Mesenchymal Phenotype of Circulating Tumor Cells in Non-Metastatic Breast Cancer

**DOI:** 10.3390/cancers11121961

**Published:** 2019-12-06

**Authors:** Marta Popeda, Tomasz Stokowy, Natalia Bednarz-Knoll, Anna Jurek, Magdalena Niemira, Agnieszka Bielska, Adam Kretowski, Leszek Kalinowski, Jolanta Szade, Aleksandra Markiewicz, Anna J. Zaczek

**Affiliations:** 1 Laboratory of Translational Oncology, Intercollegiate Faculty of Biotechnology, Medical University of Gdansk, 80-211 Gdansk, Poland; marta.popeda@gumed.edu.pl (M.P.); nbk@gumed.edu.pl (N.B.-K.); annajurek@gumed.edu.pl (A.J.); aleksandra.markiewicz@gmail.com (A.M.); 2 Department of Clinical Science, University of Bergen, 5021 Bergen, Norway; tomasz.stokowy@k2.uib.no; 3 Clinical Research Centre, Medical University of Bialystok, 15-276 Bialystok, Poland; magdalena.niemira@umb.edu.pl (M.N.); agnieszka.bielska@umb.edu.pl (A.B.); adamkretowski@wp.pl (A.K.); 4 Department of Medical Laboratory Diagnostics and Central Bank of Frozen Tissues & Genetic Specimens, Medical University of Gdansk, 80-211 Gdansk, Poland; lekal@gumed.edu.pl; 5 Biobanking and Biomolecular Resources Research Infrastructure Poland (BBMRI.PL), 80-211 Gdansk, Poland; 6 Department of Pathomorphology, Medical University of Gdansk, 80-211 Gdansk, Poland; jszade@gumed.edu.pl

**Keywords:** breast cancer, circulating tumor cells, epithelial–mesenchymal transition, NF-kappa B signaling, type I interferons, tumor microenvironment, immune-related transcriptome

## Abstract

The role of circulating tumor cells (CTCs), tumor microenvironment (TME), and the immune system in the formation of metastasis is evident, yet the details of their interactions remain unknown. This study aimed at exploring the immunotranscriptome of primary tumors associated with the status of CTCs in breast cancer (BCa) patients. The expression of 730 immune-related genes in formalin-fixed paraffin-embedded samples was analyzed using the multigenomic NanoString technology and correlated with the presence and the phenotype of CTCs. Upregulation of 37 genes and downregulation of 1 gene were observed in patients characterized by a mesenchymal phenotype of CTCs when compared to patients with epithelial CTCs. The upregulated genes were involved in NF-kappa B signaling and in the production of type I interferons. The clinical significance of the differentially expressed genes was evaluated using The Cancer Genome Atlas (TCGA) data of a breast invasive carcinoma (BRCA) cohort. Five of the upregulated genes—*PSMD7*, *C2, IFNAR1*, *CD84*, and *CYLD*—were independent prognostic factors in terms of overall and disease-free survival. To conclude, our data identify a group of genes that are upregulated in BCa patients with mesenchymal CTCs and reveal their prognostic potential, thus indicating that they merit further investigation.

## 1. Introduction

Distant metastases account for most of cancer-related deaths. Yet, fundamental questions regarding mechanisms that promote or inhibit the formation of metastasis still remain unanswered. It is evident that in breast cancer (BCa), tumor cell dissemination occurs already at early stages of the disease [1,2], with circulating tumor cells (CTCs) being direct initiators of metastasis [3]. With current analytic methods, CTCs are detectable in the peripheral blood, and their presence is considered an adverse prognostic factor in numerous solid tumors, including BCa [4,5,6,7,8,9,10,11]. Tumor dissemination is known to be facilitated by the process of epithelial–mesenchymal transition (EMT) [12]. CTCs are shown to possess phenotypic plasticity that relates to their ability to display various EMT states in the circulation [13,14,15], with either the mesenchymal [16] or the intermediate [17] phenotype associated with increased tumor-initiating ability. The presence of mesenchymal CTCs is associated with disease progression and worse prognosis for metastatic BCa [18,19,20] and even operable breast cancer patients [21,22,23]. In fact, in metastatic BCa patients, the expression of mesenchymal markers is higher than in early-stage BCa patients [24,25] and correlates with lymph node involvement [26], suggesting that the EMT phenotype is directly related to the metastatic potential of CTCs.

The ability to evade the immune system is one of the hallmarks of cancer [27,28]. Tumor cells use multiple strategies to escape immune surveillance, mainly avoiding immune recognition and instigating an immunosuppressive microenvironment [29]. The interactions between cancer cells and their microenvironment (tumor microenvironment, TME) are involved in tumor dissemination. TME is assumed to modulate the capability of CTCs to evade the innate immune response and CTCs survival [30,31]. We showed that absence of ALDH1-positive stromal cells correlates with the presence of disseminated tumor cells (DTCs) in the bone marrow of BCa patients [32]. Nevertheless, the precise profile of immune-related factors influencing tumor dissemination, in particular the presence and phenotype of CTCs, is scarcely known. Therefore, the current study aimed at exploring the association between the immunotranscriptome of primary breast tumors and the status of CTCs. We hypothesized that the presence and the EMT phenotype of CTCs are connected with a specific immune-related gene signature of primary tumors. To verify this hypothesis, we evaluated the expression of 730 immune-related genes in primary tumors of BCa patients with well-described molecular and clinicopathological features, including CTCs presence and phenotype.

## 2. Results

### 2.1. Expression of Immune-Related Genes within Primary Tumours Correlated with the Phenotype of CTCs

To investigate the immune transcriptome associated with each phenotype of CTCs, we applied NanoString multigene expression analysis to samples of primary breast tumors. We observed that the mesenchymal phenotype of CTCs (*n* = 9) was associated with the upregulation of 37 genes and the downregulation of 1 gene in primary tumors (*p*-value ≤ 0.05, false discovery rate (FDR) ≤ 0.2) when compared to the epithelial phenotype of CTCs (*n* = 14) (Table 1, Figure S1; all results in Table S1). Due to the limited number of patients included in our study, we employed the conservative FDR method of multiple testing correction.

Our aim was also to explore the association between the expression of immune-related genes within the primary tumors and the overall presence of CTCs. We found no statistically significant differences between the primary tumors’ immunotranscriptomes in relation to patients’ CTC status (positive vs. negative).

### 2.2. The Mesenchymal Phenotype of CTCs Is Associated with the Upregulation of Genes Involved in NF-kappa B Signalling and Type I Interferons Production in Matched Primary Tumours

**Figure 1:**
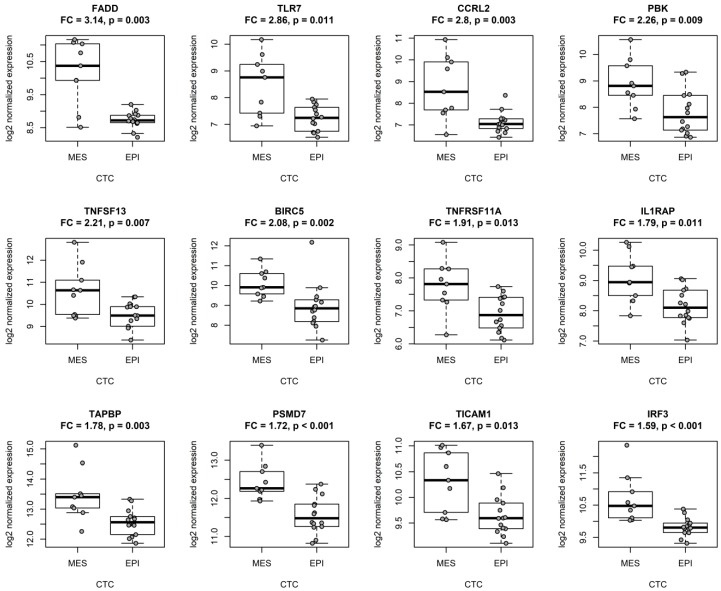
Genes implicated in NF-kappa B signaling were upregulated in primary tumors of breast cancer patients with mesenchymal CTCs (MES, *n* = 9) when compared to patients with epithelial CTCs (EPI, *n* = 14); the top 12 upregulated genes are presented. Gene expression depicted as number of counts of each probe and normalized to the four most stable reference genes (*ABCF1*, *EDC3*, *HDAC3*, and *CNOT4*); FC calculated on the basis of the median normalized counts of the probe in each group; differences in median normalized counts between groups analyzed with the Mann–Whitney U test (*p*); the bars correspond to the interquartile range (IQR), the whiskers cover 1.5 IQR from the median.

Here, we observed that multiple genes differentially expressed in patients with epithelial and mesenchymal CTC phenotypes (Table 1) play a role in the NF-kappa B signaling pathway. Consequently, we decided to interrogate this link more carefully. We applied the Functional Annotation Tool by DAVID 6.8 [33,34] to associate the selected genes with specific functional annotations. Genes upregulated in tumors with mesenchymal CTCs were generally involved in the activation and regulation of immune response (Table S2). Interestingly, 15 out of 37 upregulated genes (*FADD*, *TLR7*, *TNFRSF11A*, *IL1RAP*, *PSMD7*, *TICAM1*, *IRF3*, *BCL10*, *IKBKE*, *TRAF6*, *RELA*, *IKBKG*, *TBK1*, *PSMB10*, and *CYLD*) were implicated in the regulation of NF-kappa B signaling and activity (GO:0043122, GO:0051092, and GO:0038061). A literature search provided a number of links between other 11 differentially expressed genes (*CCRL2*, *PBK*, *TNFSF13*, *BIRC5*, *TAPBP*, *ELK1*, *STAT6*, *ATG10*, *IFNAR1*, *CCND3*, and *MAP2K1*) and the NF-kappa B pathway (top 12 genes depicted in Figure 1).

Analysis of Gene Ontology also revealed that nine of the upregulated genes regulate the production of type I interferons (GO:0032479; *TLR7*, *TICAM1*, *IRF3*, *IKBKE*, *RELA*, *TBK1*, *STAT6*, *CYLD*, and *IFNAR1*), with a particular role in the stimulation of interferon beta (GO:0032728; *TLR7*, *TICAM1*, *IRF3*, *TBK1*, and *IFNAR1*).

### 2.3. Immune-Related Genes Connected with the Mesenchymal Phenotype of CTCs Are Potent Negative Prognostic Factors in Breast Cancer

**Figure 2:**
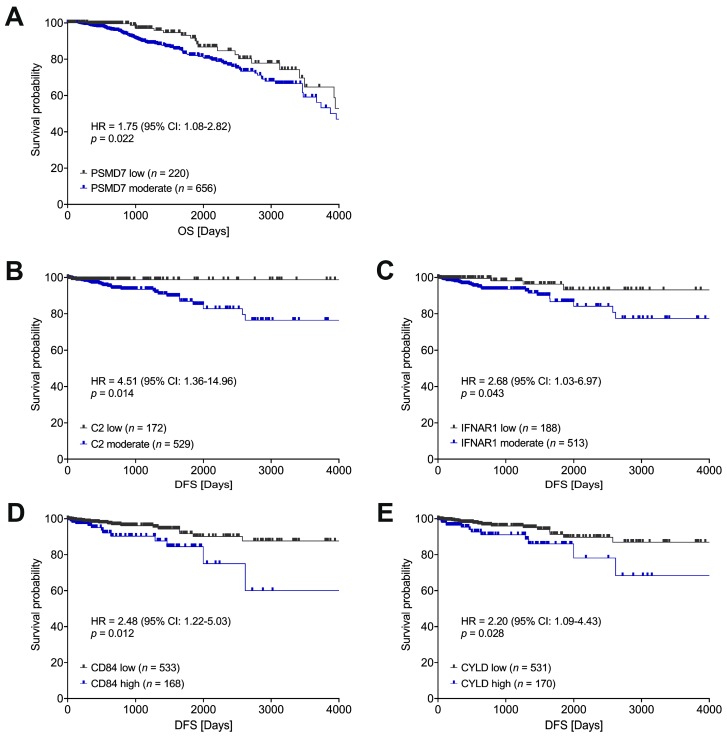
Genes associated with the mesenchymal phenotype of CTCs had a negative impact on survival (**A**) and recurrence (**B**–**E**) in patients in a BRCA cohort of The Cancer Genome Atlas (TCGA). Low/moderate status of gene expression relative to the first quartile (Q1); low/high status of gene expression relative to the third quartile (Q3); hazard ratios (HR) with 95% confidence intervals (95% CI) computed using Cox proportional hazards regression; OS: overall survival, DFS: disease-free survival.

We have previously demonstrated that the mesenchymal phenotype of CTCs correlates with a poor prognosis in breast cancer patients [21]. Consequently, we decided to evaluate the prognostic significance of the immune-related genes that we found significantly up- or downregulated in primary tumors of BCa patients with mesenchymal CTCs. To this end, we turned to The Cancer Genome Atlas (TCGA) database and analyzed the available RNA-seq data on gene expression in a breast invasive carcinoma (BRCA) cohort (*n* = 877) [35,36]. Five out of 38 genes (*PSMD7*, *C2*, *IFNAR1, CD84*, and *CYLD*) associated with the mesenchymal phenotype of CTCs demonstrated a negative prognostic impact in the TCGA cohort. Moderate (higher than the first quartile, Q1 in Table S3) expression of *PSMD7* correlated with shorter overall survival (OS) in comparison to low expression of *PSMD7* in primary tumors (HR = 1.75, 95% CI: 1.08–2.82, *p* = 0.022; Figure 2A). A higher risk of recurrence was observed for tumors with moderate (higher than the first quartile, Q1 in Table S3) expression of *C2* (HR = 4.51, 95% CI: 1.36–14.96, *p* = 0.014; Figure 2B) and *IFNAR1* (HR = 2.68, 95% CI: 1.03–6.97, *p* = 0.043; Figure 2C) in comparison to tumors with low expression of these genes; high (higher than the third quartile, Q3 in Table S3) expression of *CD84* (HR = 2.48, 95% CI: 1.22–5.03, *p* = 0.012; Figure 2D) and *CYLD* (HR = 2.20, 95% CI: 1.09–4.43, *p* = 0.028; Figure 2E) was also linked to shorter disease-free survival (DFS) in comparison to low expression of these genes in the primary tumors. Multivariate analysis including the clinical stage confirmed the significance of the aforementioned genes as independent prognostic factors (Table S3).

## 3. Discussion

The knowledge about immune signatures related to tumor dissemination is still limited. Our current study aimed to identify the immunotranscriptomic profiles of primary tumors associated with the presence of CTCs and the CTCs phenotype in non-metastatic BCa patients.

**Figure 3:**
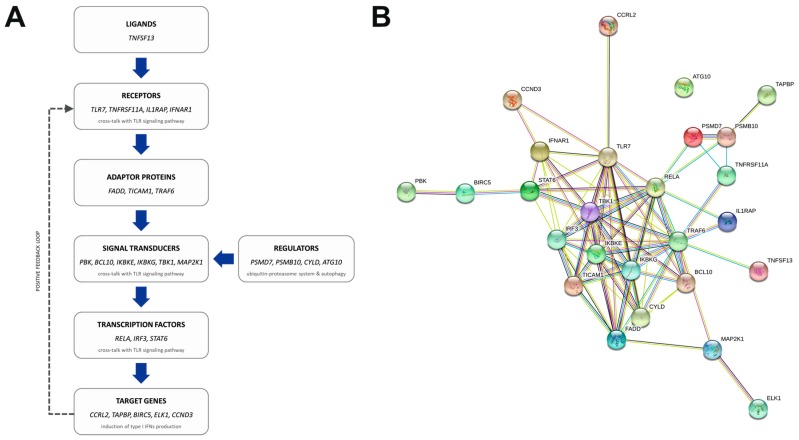
Genes upregulated in the primary tumors of patients with mesenchymal CTCs function at various levels of the NF-kappa B signaling pathway (**A**), within a complex network of interactions (**B**); image depicting a protein–protein association network, generated using the STRING tool; edge (line) coloring defines the type of interaction: blue—from curated databases, pink—experimentally determined, green—gene neighborhood, red—gene fusions, dark blue—gene co-occurrence, yellow—text mining, black—co-expression, violet—protein homology.

Our data revealed that 38 genes were differentially expressed in the primary tumors of patients with mesenchymal CTCs when compared to patients with epithelial CTCs. Intriguingly, we did not observe any statistically significant difference between primary tumor transcriptomes when comparing patients according to the presence or absence of CTCs in the circulation. The compared groups (CTC-positive and CTC-negative) were not biased in terms of any clinicopathological parameter (Table S4); hence, we believe that the observed difference in the activation of dissemination at BCa tumors may be cell context-dependent and definitely requires a more thorough analysis. On the other hand, our results demonstrate a substantial connection between the mesenchymal phenotype of CTCs and the NF-kappa B pathway. According to the NanoString gene expression assay and a literature search, 26 out of the 37 genes upregulated in mesenchymal-CTC patients in comparison to epithelial-CTC patients are implicated in NF-kappa B signaling at various levels of the transduction pathway (Figure 3A) and demonstrate a complex network of interactions at the protein level (Figure 3B). Importantly, the enrichment of NF-kappa B-related transcripts was consistently observed when we applied a stricter gene inclusion criteria and limited the analysis to a set of 330 genes with the highest expression (log2 mean count of a gene in all samples >9; Table S5).

NF-kappa B signaling is a potent regulator of numerous vital physiological processes, including survival, inflammation, and immune responses [37]. The activation of the pathway is mediated by numeros receptors. Our enriched set (Table 1) includes genes encoding both specific ligands (APRIL (*TNFSF13*)) and receptors (*TLR7*, *TNFRSF11A*, *IL1RAP*, and *IFNAR1*), as well as universal adaptor proteins (*FADD*, *TICAM1*, and *TRAF6*) that facilitate the transduction of the signal from the receptors in the cell membrane to the effectors in the nucleus. Namely, we observed the upregulation of transducers involved in the canonical cascade (*BCL10* and *IKBKG*) as well as in Toll-like receptor-mediated activation of NF-kappa B signaling (*PBK*, *IKBKE*, and *TBK1*). Moreover, the enhanced expression of *MAP2K1* gene points to the possible role of ERK-mediated stimulation of NF-kappa B signaling in tumors with mesenchymal CTCs [38]. 

On the other hand, the activity of NF-kappa B is known to be regulated by the proteasome and ubiquitin-mediated proteolysis. Here, we report the upregulation of genes that are implicated in the ubiquitin-proteasome system (*PSMD7, PSMB10*, and *CYLD*) [39] and the autophagy cascade (*ATG10*) [40,41,42]. Eventually, we observed an increased expression of one the subunits of the NF-kappa B transcription factor—p65 (*RELA*), as well as of two other co-operating transcription factors (*IRF3* and *STAT6*). The enhanced signaling resulted in the upregulation of five target genes—*CCRL2*, *TAPBP*, *BIRC5*, *ELK1*, and *CCND3*.

The NF-kappa B pathway is a well-known driver of EMT during both embryonic and tumor development [37]. In general, a constant stimulation of this pathway in cancer cells results in abnormal proliferation and differentiation, enhanced metastasis, and treatment resistance [43]. In breast cancer, NF-kappa B directly regulates the transcription of genes encoding EMT-inducing transcription factors [44]. In fact, the increased expression of NF-kappa B is a common feature of breast cancer cell lines and tissues, correlating with intensified activation of both the canonical and the non-canonical pathway [45,46,47]. What is more, several reports point to an interesting association between NF-kappa B and HER2 [47,48,49], with evidence for predominant NF-kappa B activation in ER−/HER2+ breast tumors [45,49].

Our data revealed another interesting pattern of enrichment, with upregulation of several NF-kappa B-related genes that are particularly involved in the positive regulation of type I interferon production (Table S2). The cross-talk between NF-kappa B and Toll-like receptors (TLR)-mediated signaling results in an increased pro-inflammatory response that is additionally enhanced in an autocrine and paracrine manner by a positive feedback loop (Figure 3A) [50,51].

Noteworthy, among the NF-kappa B-unrelated genes, we found markers of platelet activation (*CD63* and *CD84*) [52,53], which is in line with literature reports on the co-operation between platelets and CTCs in the induction of EMT and metastasis formation [54,55]. Of note, tumor dissemination may also be supported by other populations of cells within the intratumoral stroma. The elevated NF-kappa B activity may result from increased release of pro-inflammatory cytokines by macrophages at the tumor site [56,57,58]. In fact, NF-kappa B seems to be involved in the polarization of tumor-associated macrophages [57]. We have previously reported the negative prognostic significance of CTCs of mesenchymal phenotype in BCa patients [21]. Due to the low number of patients in this cohort, in the current study we analyzed the impact of genes linked with mesenchymal CTCs in TCGA BCa cohort. In fact, in TCGA data 5 out of the 38 genes of our interest were associated with worse prognosis (overall survival or risk of recurrence), namely, *PSMD7*, *C2*, *IFNAR1*, *CD84*, and *CYLD*. None of the corresponding proteins is currently included in the routine histopathology for breast tumor, thus they need to be validated at the protein level in a large cohort of patients in order to prove their clinical importance and diagnostic applicability.

## 4. Materials and Methods

### 4.1. Patients

The study group consisted of 35 breast cancer patients staged I–III, who had undergone surgical treatment at the Medical University Hospital in Gdansk between April 2011 and May 2013. The study was approved by the Ethical Committee of the Medical University of Gdansk (NKBBN 94/2017), and informed consent was collected from all participants. Patients were characterized by different clinicopathological parameters (Table S4), with particular focus on CTC status—negative (*n* = 12) or positive (*n* = 23)—and molecular phenotype of CTCs—epithelial (*n* = 14) or mesenchymal (*n* = 9)—as described previously [26].

### 4.2. RNA Extraction

RNA was extracted from formalin-fixed paraffin-embedded (FFPE) primary breast tumor samples (four 10 µm-thick, unstained FFPE sections per patient) using the RNeasy Mini Kit (Qiagen, Hilden, Germany) according to the manufacturer’s protocol. RNA concentration and purity were determined using NanoDrop 1000 spectrophotometer (Thermo Scientific, Wilmington, DE, USA). RNA integrity was assessed using Agilent 2100 Bioanalyzer (Agilent Technologies, Santa Clara, CA, USA) with Agilent RNA 6000 Pico Kit (Agilent Technologies).

### 4.3. nCounter Gene Expression Assay

Extracted RNA (4 µl) was pre-amplified using the nCounter Low RNA Input Kit (NanoString Technologies, Seattle, WA, USA) with the dedicated Primer Pool covering the sequences of 730 immune-related genes included in the nCounter PanCancer Immune Profiling Panel (NanoString Technologies). Pre-amplified samples were analyzed using the NanoString nCounter Analysis System (NanoString Technologies) according to the manufacturer’s procedures for hybridization, detection, and scanning.

### 4.4. Data Analysis

For each tumor sample analyzed with the NanoString technology, the background level was estimated using the mean plus 2 standard deviations of the counts of the negative control probes included in the assay. Data were normalized using the geometric mean of the positive controls included in the assay and 4 most stably expressed housekeeping genes included in the PanCancer Immune Profiling Panel—*ABCF1*, *EDC3*, *HDAC3*, and *CNOT4*—(expression stability assessed with NormFinder, SD range 173.5–228.4 counts). Background thresholding and normalization were performed using nSolver 4.0 software (NanoString Technologies).

Low-expression genes (log2 mean count of a gene in all samples <6) were excluded, leaving 584 genes for further analysis. Subsequently, the genes differentiating each CTC status were selected on the basis of fold change in comparison to the control; fold change was calculated on basis of the median normalized counts of the probe in each group. The following comparisons were performed: CTC-positive vs. CTC-negative; CTC-epithelial vs. CTC-negative; CTC-mesenchymal vs. CTC-negative; CTC-mesenchymal vs. CTC-epithelial. Genes with FC > 1 were considered upregulated; genes with FC < 1 were considered downregulated.

Data were analyzed using the R statistical computing environment (3.6.1) [59]. Differences in gene expression between groups were analyzed using the Mann–Whitney U test with Benjamini–Hochberg correction for multiple comparisons; *p*-values ≤ 0.05 and FDR values ≤ 0.2 were considered statistically significant.

For the differing genes, gene ontology was analyzed using the Functional Annotation Tool by DAVID Bioinformatics Resources 6.8 [33,34]. EASE Score, a modified Fisher exact *p*-value, was used to assess gene enrichment. Multiple testing was corrected using FDR correction.

For the NF-kappa B-related genes, a protein–protein association network was depicted using STRING v11 [60].

### 4.5. Survival Analysis in TCGA Cohort

RNA-seq (RNASeqV2, RSEM_ normalized) and clinical data of BRCA cohort were obtained from TCGA portal [35,36] (data status of 28 January, 2016). The group was limited to T1-3M0 patients, and records with missing clinical or expression values were excluded, leaving 877 out of 1098 BCa patients for the analysis. OS was defined according to the “days_to_death” variable for survival time and the “vital_status” variable for event; DFS was defined according to the “days_to_last_follow-up” variable for survival time and the “person_neoplasm_cancer_status” variable for event. For the genes of interest, low/moderate status of gene expression was determined according to the 1st quartile (Q1) cut-off, while low/high status of gene expression was determined according to the 3rd quartile (Q3) cut-off. For each gene, the expression status (low vs. moderate; low vs. high) was tested in both univariate and multivariate analyses including the clinical stage. Hazard ratios (HR) with 95% confidence intervals (95% CI) were computed using Cox proportional hazards regression using the R statistical computing environment (3.6.1) [59].

## 5. Conclusions

To summarize, this study points to the potential link between the expression of immune-related genes in cells within the primary tumor and the EMT state of circulating tumor cells. Increased NF-kappa B signaling-related signatures in the tumor mass might possibly promote EMT in CTCs, thus contributing to their more aggressive phenotype and worse patient prognosis. It merits further investigation whether such effect is due to the action of cancer cells or that of normal cells in the surrounding TME. The potential prognostic relevance of selected genes associated with mesenchymal CTCs is promising and deserves further validation.

## Figures and Tables

**Table 1:** Genes up- and downregulated in primary tumors of patients with mesenchymal circulating tumor cells (CTCs) (compared to patients with epithelial CTCs).

Gene Symbol	Gene Name	FC	*p*-Value	FDR
*LAIR2*	Leukocyte-associated immunoglobulin-like receptor 2	3.19	0.007	0.144
*FADD*	Fas-associated via death domain	3.14	0.003	0.082
*TLR7*	Toll-like receptor 7	2.86	0.011	0.172
*CCRL2*	C–C motif chemokine receptor-like 2	2.80	0.003	0.087
*PBK*	PDZ-binding kinase	2.26	0.009	0.161
*CD3EAP*	CD3e molecule-associated protein	2.25	0.001	0.063
*CD84*	CD84 molecule	2.22	0.003	0.087
*TNFSF13*	TNF superfamily member 13	2.21	0.007	0.144
*BIRC5*	Baculoviral IAP repeat-containing 5	2.08	0.002	0.082
*KLRC1*	Killer cell lectin-like receptor C1	1.98	0.001	0.063
*CD63*	CD63 molecule	1.93	0.003	0.082
*TNFRSF11A*	TNF receptor superfamily member 11a	1.91	0.013	0.199
*C2*	Complement C2	1.85	0.001	0.069
*IL1RAP*	Interleukin 1 receptor accessory protein	1.79	0.011	0.172
*TAPBP*	TAP-binding protein	1.78	0.003	0.082
*NUP107*	nucleoporin 107	1.78	0.002	0.082
*PSMD7*	Proteasome 26S subunit, non-ATPase 7	1.72	<0.001	0.063
*TICAM1*	Toll-like receptor adaptor molecule 1	1.67	0.013	0.199
*ICAM3*	Intercellular adhesion molecule 3	1.63	0.009	0.161
*IRF3*	Interferon regulatory factor 3	1.59	<0.001	0.048
*BCL10*	BCL10 immune signaling adaptor	1.56	0.001	0.069
*IKBKE*	Inhibitor of nuclear factor kappa B kinase subunit epsilon	1.55	0.011	0.172
*ELK1*	ETS transcription factor ELK1	1.55	0.001	0.069
*TRAF6*	TNF receptor-associated factor 6	1.53	0.003	0.087
*RELA*	RELA proto-oncogene, NF-kB subunit	1.52	0.003	0.082
*IKBKG*	Inhibitor of nuclear factor kappa B kinase regulatory subunit gamma	1.49	0.003	0.087
*TBK1*	TANK-binding kinase 1	1.46	0.001	0.063
*STAT6*	Signal transducer and activator of transcription 6	1.40	0.004	0.106
*ATG10*	Autophagy-related 10	1.40	0.007	0.144
*CD74*	CD74 molecule	1.38	0.009	0.161
*ICOSLG*	Inducible T cell costimulator ligand	1.38	0.002	0.082
*FCGR2A*	Fc fragment of IgG receptor IIa	1.37	0.007	0.144
*PSMB10*	Proteasome 20S subunit beta 10	1.36	0.011	0.172
*CYLD*	CYLD lysine 63 deubiquitinase	1.36	0.011	0.172
*IFNAR1*	Interferon alpha and beta receptor subunit 1	1.35	0.001	0.069
*CCND3*	Cyclin D3	1.30	0.002	0.082
*MAP2K1*	Mitogen-activated protein kinase kinase 1	1.25	<0.001	0.027
*MERTK*	MER proto-oncogene, tyrosine kinase	0.68	0.003	0.082

Fold change (FC) based on median normalized counts of the probe in each group; differences in median normalized counts between groups analyzed with Mann–Whitney U test (*p*-value) with Benjamini–Hochberg correction for multiple comparisons (false discovery rate, FDR); only statistically significant results are presented; gene names according to HUGO Gene Nomenclature.

## Supplementary Materials

The following are available online at https://www.mdpi.com/2072-6694/11/12/1961/s1, Table S1: Results of gene expression analysis according to each CTC status or phenotype, Table S2: List of GO terms enriched in the genes upregulated in mesenchymal-CTC patients, Table S3: Results of multivariate survival analysis depending on the expression of genes up- and downregulated in mesenchymal-CTC patients; low/moderate status of gene expression relative to the 1st quartile (Q1); low/high status of gene expression relative to the 3rd quartile (Q3); hazard ratios (HR) with 95% confidence intervals (95% CI) computed using Cox proportional hazards regression; significant results are highlighted in green, Table S4: Clinicopathological characteristics of the patients, Table S5: List of genes upregulated in the primary tumors of patients with mesenchymal CTCs (compared to patients with epithelial CTCs), computed for a set of 330 high-expression genes (log2 mean count of a gene in all samples > 9), Figure S1: Expression of genes up- and downregulated in primary tumors of patients with mesenchymal CTCs (MES, *n* = 9) and patients with epithelial CTCs (EPI, *n* = 14); expression depicted as number of counts of each probe and normalized to the 4 most stable reference genes (*ABCF1*, *EDC3*, *HDAC3*, and *CNOT4*); fold change (FC) based on the median normalized counts of the probe in each group; differences in median normalized counts between groups analyzed with the Mann–Whitney U test (*p*); bars correspond to IQR, whiskers cover 1.5 IQR from the median.
